# Comparison of normal hindlimb lymphatic systems in rats with detours present after lymphatic flow blockage

**DOI:** 10.1371/journal.pone.0260404

**Published:** 2021-12-13

**Authors:** Yuiko Suzuki, Yukari Nakajima, Toshio Nakatani, Mayumi Okuwa, Junko Sugama

**Affiliations:** 1 Division of Health Sciences, Graduate School of Medical Sciences, Kanazawa University, Ishikawa, Japan; 2 Faculty of Health Sciences, Komatsu University, Ishikawa, Japan; 3 Faculty of Health Sciences, Institute of Medical, Pharmaceutical and Health Sciences, Kanazawa University, Ishikawa, Japan; 4 Research Center for Implementation Nursing Science Initiative, School of Health Sciences, Fujita Health University, Aichi, Japan; University of Minnesota Medical School, UNITED STATES

## Abstract

In the present study, we aimed to identify the normal hindlimb lymphatic systems in rats and compare them with the detours after lymphatic flow blockage. The lymphatic systems of the hindlimbs of normal rats were investigated via lymphography using a near-infrared fluorescence imaging system. The lymphatic vessels were stained using Evans Blue. The lymphatic flow was blocked through lymphatic vessel ligation combined with inguinal and popliteal lymph node dissection. Detours that appeared after 30 days were visualized using lymphography and immunostaining with anti-podoplanin antibodies. Three main results were obtained in the present study. First, the deep medial system, the superficial medial system, a connection between the superficial and deep medial lymphatic systems, and the superficial lateral system, were elucidated. Second, three types of detours, namely the detour of the lateral abdomen, the detour to the lymphatic vessel near the midline of the abdomen, and the detour to the contralateral inguinal lymph node, were identified after lymphatic flow blockage. Lastly, detours were located in the fatty layer above the panniculus carnosus muscle and their lumina were wide. The histology suggested that the detour was a pre-collecting lymphatic vessel. Lymphatic routes in the rat hindlimbs after lymphatic flow blockage were different from those of the normal rat lymphatic system. It was suggested that the detour is a pre-collecting lymphatic vessel and that encouraging its development may be a new method of simple lymphatic drainage.

## Introduction

Secondary lymphedema is a chronic disease that is characterized by tissue swelling due to excess lymphatic fluid retention in the interstitial spaces. Breast cancer-related lymphedema occurs after breast cancer treatment, such as the dissection of lymph nodes (LNs) in cancer resection or radiation therapy [1, 2]. Depending on the surgery and treatment applied, approximately 11%–57% of the patients with breast cancer develop lymphedema [3]. Lymphedema can significantly reduce a patient’s quality of life by limiting limb function and may be accompanied by a remarkable change in appearance [4, 5].

Currently, there is no curative treatment for lymphedema. Complete decongestive therapy is considered the gold-standard treatment for managing lymphedema and includes two phases: reduction and maintenance [6]. In the maintenance phase, patients are requested to perform daily self-care, including simple lymphatic drainage (SLD), application of compression garments, physical exercises, and skincare [7]. The aim of SLD is to prevent the accumulation of lymph fluid, but a full body massage is time-consuming. Therefore, SLD makes self-care difficult and hinders social participation. Furthermore, the effectiveness of SLD is unclear owing to the paucity of information [7–9].

The lymphatic flow that occurs after lymphatic flow blockage differs from the one that occurs before surgery. Generally, once lymph is taken up by the capillary lymphatic vessels (LVs), it travels through the pre-collecting LVs to the collecting LVs in the deep dermis and subcutaneous layer. The collecting LVs are accompanied by smooth muscle cells and exist in parallel with the vascular system. In addition, lymph is collected in LVs and transported to deeper LVs [10]. Liu et al. used post-contrast magnetic resonance imaging to demonstrate varied lymphatic distribution patterns and abnormal lymph flow pathways within the limbs of patients with lymphatic circulation disorders [11]. Lymphatic drainage routes have also been shown to change, i.e., detour, in rats when the lymph flow is blocked [12, 13]. Therefore, it is thought that excess lymph can be effectively removed by directing it toward a detour. It suggests the possibility of performing localized massage.

Detours after lymphatic flow blockage have been studied using animal models. Rodent hindlimbs provide the most feasible, cost-effective, and qualified model for studying lymphatic function and repair [14]. In a rat model, lymphedema develops when the lymphatic flow is blocked by LN dissection and ligation of the LVs. However, the volume of lymphedema tends to decrease owing to the subsequent appearance of detours. For this reason, it is thought that a detour after lymphatic flow blockage is likely to facilitate the drainage of retained lymph fluid. Therefore, various types and forms of detours are being studied [15, 16]. However, in addition to these studies, a comparison between normal lymphatic pathways and detours is needed for a comprehensive understanding of the nature of detours.

The normal rat hindlimb lymphatic system is only partially known. The lymphatic system of the rat has been studied using techniques such as lymphatic staining and indocyanine green (ICG) lymphangiography. The locations of the LNs, regions of lymphatic drainage of the cutaneous areas, and some superficial and deep LVs have been identified in the rat hindlimb lymphatic system [16–18]. However, the lymphatic system of the whole hindlimb is unclear.

In the present study, we aimed to identify the normal hindlimb lymphatic systems of the rat and compare them to the detours after lymphatic flow blockage.

## Materials and methods

All experimental protocols and animal care procedures were approved by the Committee on Animal Experimentation of Kanazawa University (AP-194065) and were performed according to its guidelines. At the time of dissection, the rats were euthanized with 5% isoflurane inhalation anesthesia. The humane endpoint during the study was set at the time of postoperative wound infection. We adopted the same anatomical terms for the rat lymphatic system as those used by Suami *et al*. [17] and Tilney [18].

### Animals

Twenty Slc: Wistar rats (Sankyo Lab Service Corporation, Inc., Toyama, Japan) were used. Both male and female rats were prepared to identify sex-dependent differences in the lymphatic system. They were housed at 25.0 ± 2.0°C in an air-conditioned room, which was lit from 08:00 hours to 20:00 hours. Each rat was housed in a separate cage and was given free access to water and food. Five male and five female 20-week-old rats were used to determine the normal lymphatic pathways; six male and four female rats aged over 10 weeks were used to determine the detours after lymphatic flow blockade. Two male rats died due to bleeding during surgery. The mortality rate was 20.0%.

### Lymphology determined by near-infrared fluorescence (NIRF) imaging

ICG lymphography is a helpful technique for investigating the lymphatic system [19]. The rats were anesthetized with 2.4% isoflurane, and the concentration of the anesthetic was maintained at 1.7%–2.3%. A pde-neo NIRF imaging system (Hamamatsu Photonics, Shizuoka, Japan) was used to visualize the lymphatics; 20 μL ICG (2.5 mg/ml; Daiichi Sankyo, Tokyo, Japan) was injected into the subcutaneous tissue or the dermis to identify the hindlimb lymphatic system in the normal rats. Based on previous study, the injection points were the foot, inner ankle, outer ankle, lower leg, thigh, and tail [20]. ICG was also injected subcutaneously into the abdomen and lower abdomen to distinguish them from the hindlimb lymphatic systems. Furthermore, ICG was also injected into the dermal layer of the plantar and ankle of each rat 30 days after lymphatic flow blockade to visualize the postoperative detours. Fluorescent LVs and LNs were then examined using the NIRF imaging system.

### Lymphatic vessel examination via Evans Blue staining

A mixture of ICG and 3% w/v Evans Blue (EB; Sigma-Aldrich Japan, Tokyo, Japan) was prepared. The ratio of ICG to EB was set at 1:2. The LVs that were stained blue with EB were visualized macroscopically. The injection points were the same as those used for ICG injection. The rats were euthanized with 5% isoflurane 15–30 min after injection and dissected to examine their LVs.

### Surgical procedure for lymphatic flow blockage

LN dissection was performed using the method described by Nakajima *et al*. [20]. Details of the procedure are given below. The rats were anesthetized with 2.4% isoflurane, and the concentration of the anesthetic was maintained at 1.7%–2.3%. Next, the mixture of ICG and EB was administered to the rats to visualize the lymphatic system. Then, flunixin (5 mg/kg weight; Meiji Seika Pharma Co., Ltd., Japan) and enrofloxacin (8.5 mg/kg weight; Wako Pure Chemical Industries, Ltd., Osaka, Japan) were injected subcutaneously for analgesic and antibiotic cover, respectively. A 2-cm incision was made through the skin and abdominal muscles using surgical scissors, and the right iliac LN was excised from the abdominal cavity. First, the abdominal muscle and subsequently the skin were sutured with 6/0 nylon thread. To reduce the stress on the rats from prolonged anesthesia and laparotomy for iliac LN resection, the next LN dissection was scheduled one week later. The rats were then anesthetized and EB was administered to the lower limb and foot to visualize the inguinal and popliteal LNs. Using surgical scissors, 2.0-cm incisions were made in the inguinal regions of the rats. Subsequently, the ICG-fluorescent and EB-stained LVs were ligated with 10/0 nylon thread, and the entire inguinal LN and the surrounding adipose tissue were excised using an electric scalpel under magnification. Then, the right popliteal LN was excised using an electric scalpel. The skin was sutured with a 6/0 nylon thread. All surgeries were performed on the right hindlimb. For postoperative care, flunixin (5 mg/kg weight) was administered subcutaneously for seven days for analgesia in both surgeries. The rats were monitored daily for weight, wound condition, and health during the first week after surgery.

### Assessment of the hindlimb

Circumference and volume measurements were used to evaluate for swelling in the hindlimbs of the rats. The circumference was measured along the rat’s inguinal meridian by medical tape (Micropore, TM surgical tape, Tokyo, Japan). The volume was measured by the water displacement volumetry method (MK-101P plethysmometer, Muromachi Kikai co., Ltd, Tokyo, Japan). The hindlimbs of the rats were placed in water up to the groin. These measurements were performed before iliac LN dissection and at 3 and 30 days after inguinal and popliteal LN dissection; each measurement was performed three times by a single researcher (Y.S.) to obtain a mean value. Statistical analysis was performed using SPSS version 23. The Wilcoxon rank sum test was used to evaluate left-right differences in the circumference and volume of the hindlimbs. P-values of <0.05 was considered statistically significant.

### Immunohistochemistry

Immunostaining with anti-podoplanin antibodies was performed to investigate the lymphatic system. The locations of the LVs visualized with ICG were marked by a permanent marker on the skin. The rats were euthanized and dissected, and the necessary tissues were collected after confirming that the blue-stained LVs matched the marks made by ICG. These specimens were stapled to a plastic plate to prevent shrinkage and fixed by soaking overnight in 4% paraformaldehyde. Subsequently, the tissues were washed with phosphate-buffered saline, and soaked overnight in 10%, 20%, and 30% sucrose solutions. The removed specimens were shaped and embedded in optimal cutting temperature compounds, and frozen using isopentane (Nacalai Tesque, Kyoto, Japan). The frozen tissues were stored at -30°C until required. Using a microtome, the frozen tissues were sliced into 7-μm-thick sections, which were mounted on glass slides, dried, and washed with a mixture of phosphate-buffered saline and 0.03% Tween-20. The sections were processed with a protein-blocking solution (X0909, Dako, California, USA) and a 0.03% solution of hydrogen peroxide in methanol, and then incubated with primary antibodies, i.e., anti-rat podoplanin and monoclonal antibodies (Fujifilm Wako Pure Chemical Corporation, Osaka, Japan). Sections were then incubated with secondary antibodies conjugated to horseradish peroxidase. The secondary antibodies comprised a goat anti-mouse IgG (H+L) antibody and a horseradish peroxidase conjugate (Proteintech, Tokyo, Japan).

## Results

### Lymphatic flow elucidated by lymphography and tissue staining

The lymphatic routes were identified through ICG lymphography, EB staining, and immunostaining with the anti-podoplanin antibody. We used the same nomenclature for the hindlimb lymphatic system as that used in a previous investigation of the anatomy of the lymphatic routes in the hindlimbs of normal mice [20].

**Fig 1:**
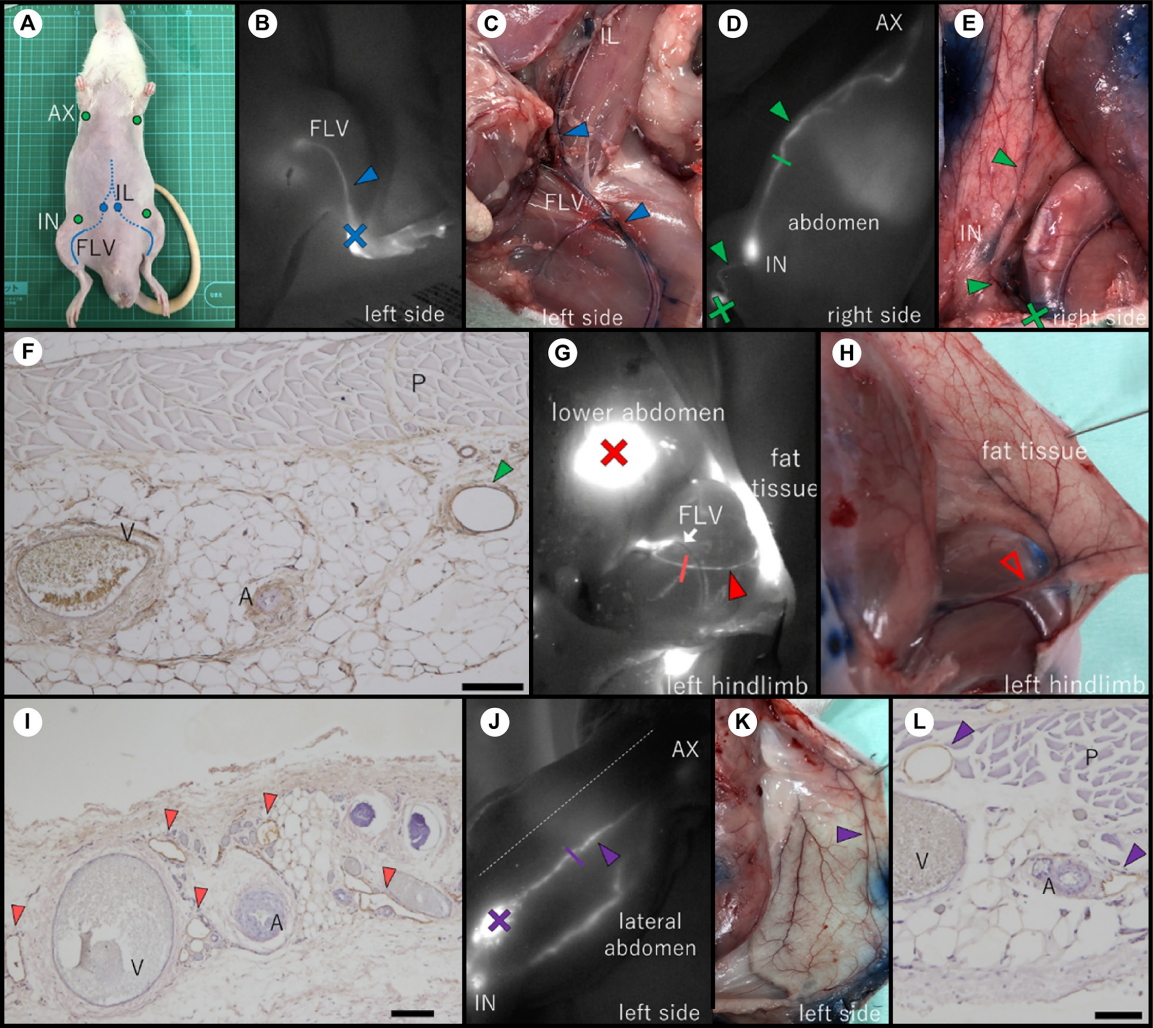
Lymphatic vessels of the ventral side of a rat. (A) LNs in the ventral side. (C), (E), (H), and (K) are the dissection pictures of the normal rat and (B), (D), (G), and (J) show the corresponding indocyanine green (ICG) images of the normal rat. (F), (I), and (L) show the results of immunostaining with the anti-podoplanin antibody. (B) ICG injected into the inner ankle caused FLV to fluoresce. (C) EB-stained image of the route identified in Fig 1B. It corresponds to route1 in Fig 3A. (D) ICG injected into the hindlimb moved through the inguinal LN to reach the axillary LN. (E) The lymphatic route in Fig 1D stained with EB was observed subcutaneously. It corresponds to route2 in Fig 3A. (F) Magnified image of the cross section at the position indicated by the green line in Fig 1D (scale bar: 200 μm). (G) ICG injected into the lower abdomen spread into the fatty layer of the inguinal region and revealed this LV. The LV started in the inguinal region and led to the FLV (white arrow). It corresponds to route3 in Fig 3A. The rat’s skin has been cut open. (H) The picture of the area where the LV in Fig 1G was identified on ICG. LV in Fig 1G could not be observed grossly by EB staining. The open red arrowhead indicates the artery paralleling ICG fluorescence. (I) Magnified image of the cross section at the position indicated by the red line in Fig 1G (scale bar: 100 μm). (J) The ICG fluorescence reached the axillary LN through a position closer to the midline than the LV seen in Fig 1D. The route was observed when ICG was injected into the abdomen and corresponds to route4 in Fig 3A. The dashed line indicates the midline. (K) Autopsy picture of the LV in Fig 1J confirmed by EB staining. (L) Magnified image of the cross section at the position indicated by the purple line in Fig 1J (scale bar: 200 μm). The closed arrowheads indicate LVs. The cross marks indicate the ICG injection points. AX, axillary LN; IN, inguinal LN; IL, iliac LN; FLV, femoral lymph vessel; A, artery; V, vein; P, panniculus carnosus muscle.

Each rat had three lymphatic systems on its ventral side (Fig 1). First, the deep medial system was identified when ICG was injected into the rat’s inner ankle. It is likely could no longer visualize the fluorescence at the inguinal area due to the increasing depth of the LV below the skin (Fig 1B). This route was connected to the iliac LN in the abdominal cavity (Fig 1C). Second, the superficial medial system was identified when ICG was injected into the outer ankle, lower leg, or thigh. The fluorescent ICG then passed through the inguinal LN and into the axillary LN (Fig 1D and 1E). Histological imaging of the marker site shows the collecting LVs (indicated by the green arrowhead) below the panniculus carnosus muscle (Fig 1F). This is a magnified tissue image of the collecting LV indicated by the green line in Fig 1D. Third, a lymphatic route was observed between the fat tissue in the inguinal region and the femoral LV (FLV). The ICG injected in the lower abdomen fluoresced the fat tissue in the inguinal region below it. The LV was observed as a single fluorescent line leading from the fat tissue in the inguinal region to the FLV when the skin at the site of ICG administration was incised (Fig 1G). It indicated a connection between the superficial and the deep medial systems. However, it was not possible to discern the connection macroscopically through EB staining (Fig 1H). Therefore, this pathway was collected using an ICG marker and the artery that paralleled this route as a landmark. Fig 1I is a magnified image of a cross section taken at the position indicated by the red line in Fig 1G. Several LVs (indicated by red arrowheads) were observed around the artery and vein. The LVs appeared as irregular brown rings. When ICG was injected into the abdomen, a fluorescent line was observed close to the midline, and this line reached the axillary LN. This LV was different from the superficial medial system (Fig 1J and 1K). In contrast, when ICG was injected into the abdomen caudal to the inguinal LNs, ICG fluorescence moved toward the inguinal LN, and then to the axillary LNs. Fig 1L is a magnified image of a cross section taken at the position indicated by the purple line in Fig 1J. Several lymphatic lumina (indicated by purple arrowheads) were observed around the blood vessels below the panniculus carnosus muscle.

**Fig 2:**
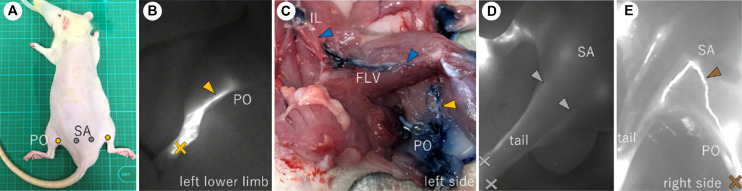
Lymphatic vessels of the dorsal side of a rat. (A) Location of the LNs of the dorsal side of a normal rat. (B), (D), (E) ICG images of the dorsal side of the hindlimb and the tail. (B) ICG injected into the footpad reached the popliteal LN. (C) Results of staining with EB. The LV leaving the popliteal LN reached the iliac LN in the abdominal cavity. The LV from the popliteal LN ran between the muscles and joined the FLV. (D) The ICG injected into the tail reached the sacral LN. (E) The connection between the popliteal LN and the sacral LN. (B), (D), (E) The crosses indicate the ICG injection points, and the arrowheads indicate the LVs. PO, popliteal LN; SA, sacral LN; FLV, femoral lymph vessel.

There was one lymphatic system involved in hindlimb lymphatic drainage on the dorsal side of each rat (Fig 2). This superficial lateral system appeared when ICG was injected into the footpad (Fig 2B). The ICG in the plantar moved towards the popliteal fossa. After passing through the popliteal LN, the LVs joined the FLV and then reached the iliac LN in the abdominal cavity (Fig 2C). The lymphatic route through the tail was confirmed by ICG injection into the tail (Fig 2D). Only 2 of the 10 rats had a connection between the popliteal and sacral LNs (Fig 2E). This route was revealed when ICG was injected into the footpad.

**Fig 3:**
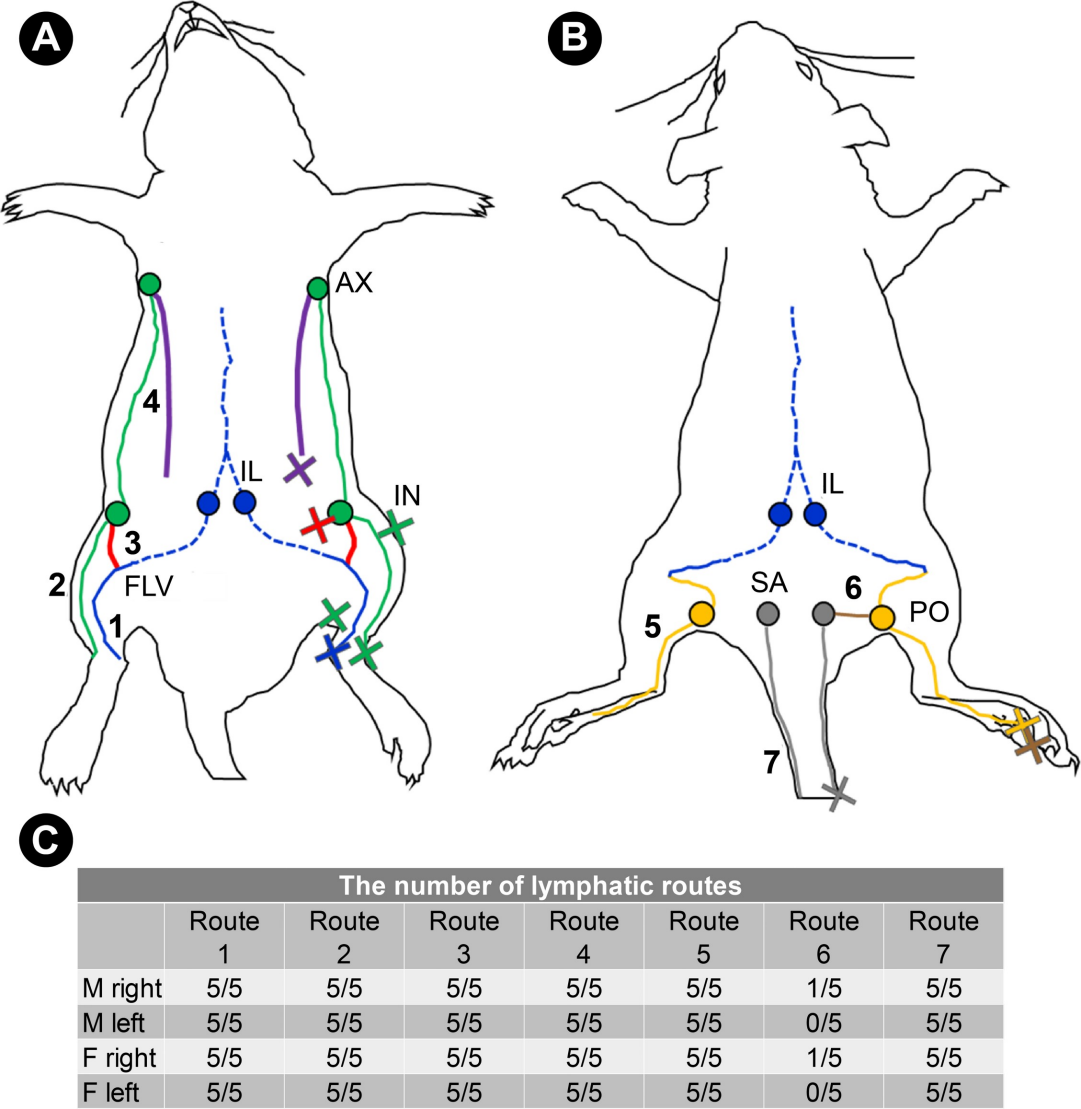
The lymphatic system in and near the hindlimb of the normal rat. (A) The lymphatic system of the ventral side. Route1, shown by the blue lines, indicates the deep medial system; route2, shown by the green lines, indicates the superficial medial system; route3, shown by the red lines, indicates a connection between the superficial and deep medial lymphatic system, and route4, shown by the purple lines, indicates lymphatics that started at the abdomen and ran into the axillary LN. (B) The lymphatic system of the dorsal side. Route5, shown by the yellow lines, indicates the superficial lateral system. The blue line indicates the FLV, and route5 was joined to it. The blue dotted lines in Fig 3A and 3B indicate the deep LVs in the abdominal cavity. Route6, shown by the brown line, indicates a connection between the popliteal LN and the sacral LN. Route7, shown by the gray lines, indicates the lymphatic route of the tail. (C) The number of lymphatic routes identified in all rats. All routes except for the connection between the popliteal LN and the sacral LN were observed in all five males and females. Cross marks indicate the injection points of ICG. Route1 was identified for inner ankle injections; route2 for outer ankle, lower limb, and thigh injections; route3 for lower abdominal injections; route4 for abdominal injections; route5 and route6 for footpad injections; and route7 for tail injections. AX, axillary LN; IN, inguinal LN; IL, iliac LN; FLV, femoral lymph vessel; PO, popliteal LN; SA, sacral LN.

Except for a connection between the popliteal and sacral LNs, there were no individual differences in the lymphatic system of the hindlimbs between the sexes. All the lymphatic systems described above were paired. The lymphatic routes in the hindlimbs of the normal rats are shown based on our observations (Fig 3C).

### Hindlimb status after lymphatic flow blockage

Before lymphatic flow blockage, there was no difference in the circumference and volume of the right and left hindlimbs. When lymph flow was blocked, 3 days later, both the circumference and volume of the right hindlimb were larger than those of the left hindlimb. However, there was no difference in the size of the right and left hindlimbs at 30 days after lymphatic flow blockage (Table 1).

**Table 1:** Circumference and volume differences between the right and left hindlimbs before and after lymphatic flow blockage.

	Right	Left	*P-*value
	(n = 8)	(n = 8)	
**Circumference (cm)**			
Before	7.5 (7.3-8.1)	7.6 (7.2-8.1)	0.731
3 days after	8.3 (7.8-8.7)	7.8 (7.3-8.5)	0.042
30 days after	7.7 (7.3-8.4)	8.1 (7.5-8.5)	0.726
**Volume (ml)**			
Before	5.5 (4.3-7.9)	5.5 (4.1-7.4)	0.400
3 days after	6.0 (4.7-9.4)	5.5 (3.9-8.5)	0.012
30 days after	5.5 (4.4-8.7)	5.5 (3.8-8.0)	0.674

Data are expressed as median (interquartile range). The Wilcoxon rank sum test was used for statistical analyses. P-values of <0.05 were considered statistically significant.

### Detours identified after lymphatic flow blockage

**Fig 4:**
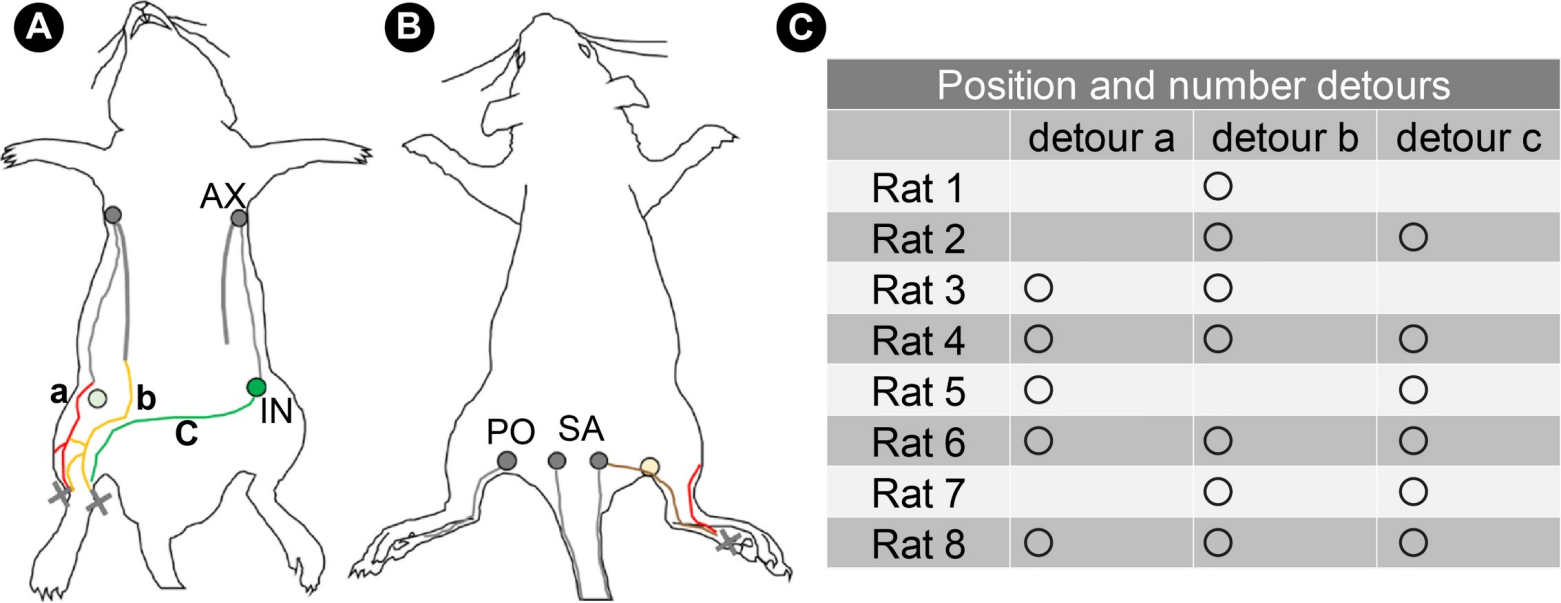
Detours identified after lymphatic flow blockage. (A) Detours: a, the detour of the lateral abdomen; b, the detour to the LV near the midline of the abdomen; c, the detour to the contralateral inguinal LN. The light green circle indicates the site of inguinal LN dissection. (B) The lymphatic route crosses the popliteal region and connects with the sacral LN without detouring. The light-yellow circle indicates the site of popliteal LN dissection. (C) The location and number of detours observed in each rat. Circles show that detours have been identified. Cross marks indicate the injection points of ICG. AX, axillary LN; IN, inguinal LN; PO, popliteal LN; SA, sacral LN.

Detours were identified in each rat 30 days after lymphatic flow blockage (S1 Fig). There were three main types of detours (Fig 4A). In addition to these detours, a route was identified on the dorsal side of the rat that penetrated the site of the popliteal LN dissection and reached the sacral LN (Fig 4B). One to three detours appeared in each rat (Fig 4C).

**Fig 5:**
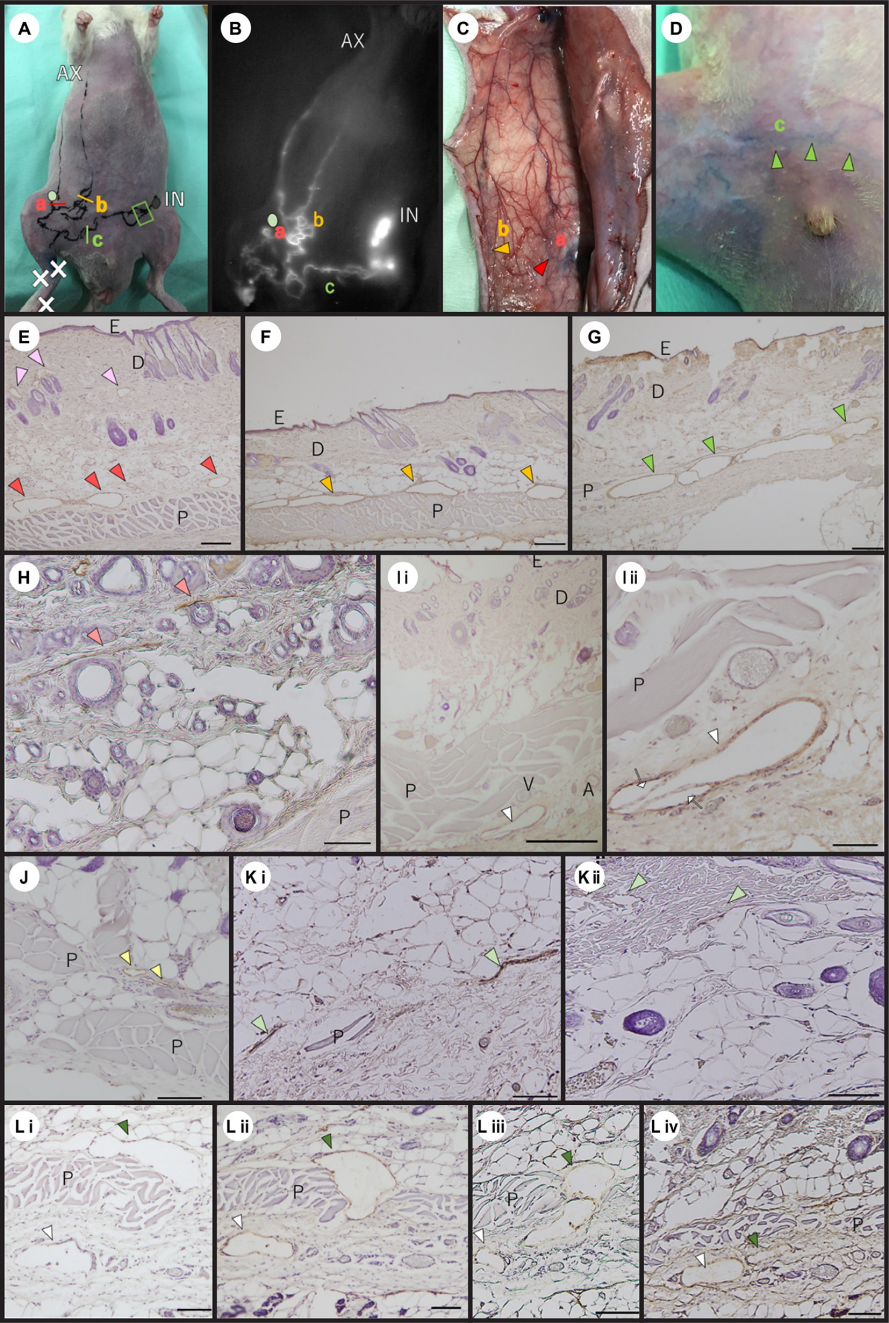
Histological characteristics of the detours. (A) A picture of the detours after lymph flow blockage. a, b, and c indicate three types of detours. The light green circle shows the position of inguinal LN dissection. (B) ICG image of detours corresponding to those indicated in Fig 5A. The light green circle shows the position of inguinal LN dissection. (E to G), (Li to Lⅳ) Immunostaining of the detour with the anti-podoplanin antibody after lymphatic flow blockage. (H to Kⅱ) Immunostaining with the anti-podoplanin antibody in normal rats. (B) Detour a of the lateral abdomen was connected to the head and caudal parts of the superficial medial system cut-off by the LN dissection. Detour b exited from the hindlimb LVs and avoided the inguinal LN dissection site, connecting the LV that started at the abdomen and ran into the axillary LN. Detour c connected to the LVs of the hindlimb and the contralateral inguinal LN. (C) An EB-stained image of detour a and detour b. The red and yellow arrowheads show each detour. (D) An EB-stained image of detour c observed from the skin surface. The green arrowheads show the detour c. (E) A high-magnification image of a cross section taken at the position indicated by the red line in Fig 5A. The red arrowheads show pre-collecting LVs with wide lumina. The pink arrowheads show capillary LVs of the dermis (scale bar: 200 μm). (F) A high-magnification image of a cross section taken at the position indicated by the yellow line in Fig 5A. The yellow arrowheads indicate pre-collecting LVs with wide lumina above the panniculus carnosus muscle (scale bar: 200 μm). (G) A high-magnification image of a cross section taken at the position indicated by the green line in Fig 5A. The green arrowheads indicate pre-collecting LVs with wide lumina below the panniculus carnosus muscle (scale bar: 200 μm). (H) The dermis and subcutaneous tissue of the normal rat corresponding to the detour sites shown in Fig 5E. The light red arrowheads indicate pre-collecting LVs (scale bar: 100 μm). (Iⅰ) The dermis and subcutaneous tissue of the normal rat corresponding to the more cephalad site of the detour a (scale bar: 500 μm). The white arrowhead shows the collecting LV. (Iⅱ) A magnified image of Fig 5Iⅰ (scale bar: 100 μm). The white arrowhead shows the collecting LV. The white arrows indicate the valves. (J) The subcutaneous tissue of the normal rat corresponding to the sites of detour b. The light-yellow arrowheads indicate pre-collecting LVs above the panniculus carnosus muscle (scale bar: 100 μm). (Kⅰ) The tissue of normal rats corresponding to the sites of detour c. The light green arrowheads indicate pre-collecting LVs near the panniculus carnosus muscle (scale bar: 200 μm). (Kⅱ) The capillary LVs of the normal dermis. The light green arrowheads indicate LVs (scale bar: 100 μm). (Lⅰ to Lⅳ) A histological image of the detour c to the contralateral inguinal LN. Serial sections were taken from the area enclosed by the green line in Fig 5A. The deep green arrowheads indicate the same pre-collecting LV. The white arrowheads show the same collecting LV. (Lⅰ) Most distant from the contralateral inguinal LN. The pre-collecting LV is located above the panniculus carnosus muscle (scale bar: 100 μm). (Lⅱ) The pre-collecting LV is penetrating the panniculus carnosus muscle (scale bar: 100 μm). (Lⅲ) The image is closer to the contralateral inguinal LN (scale bar: 100 μm). (Lⅳ) An image of the most proximal contralateral inguinal LN. The pre-collecting LV has moved below the panniculus carnosus muscle and is approaching the collecting LV (scale bar: 200 μm). AX, axillary LN; IN, inguinal LN; A, artery; V, vein; D, dermis; E, epidermis; P, panniculus carnosus muscle.

The first detour connected the cranial and caudal parts of the superficial medial system cut by the LN dissection: detour a (Fig 5A–5C). This first detour was confirmed in five of the eight rats (Fig 4C). The LVs with wide lumina above the panniculus carnosus muscle (indicated by the red arrowheads) were revealed by immunostaining with the anti-podoplanin antibody (Fig 5E). Linear LVs were identified in the dermal and subcutaneous tissues of the normal rat at the same site as this detour (Fig 5H). In normal rats, in addition, collecting LVs were identified below the panniculus carnosus muscle, corresponding to LVs more cephalad than the detour (Fig 5Ii and 5Iii).

The second detour was connected to the LVs in the hindlimb and the LV near the midline: detour b (Fig 5B and 5C). This detour was found in seven of the eight rats. A magnified image of a cross section taken at the position indicated by the yellow line in Fig 5A shows LVs with wide lumina (indicated by yellow arrowheads) above the panniculus carnosus muscle (Fig 5F). The LVs at the same site in the normal rat had narrow lumina (Fig 5J).

The third detour passed over the pubis and reached the contralateral inguinal LN: detour c (Fig 5B–5D). The fluorescent ICG reached the contralateral inguinal LN without interruption. This detour was confirmed in six of the eight rats. In the magnified image of the cross section taken at the position indicated by the green line in Fig 5A, an LV with a wide lumen was observed (Fig 5G). However, linear LVs were identified near the panniculus carnosus muscle at the same site in the normal rats (Fig 5Ki). Linear LVs were also present in the dermal layer in the same tissues (Fig 5Kii). In the histology near the contralateral inguinal LN in detour c, the detour passing through the panniculus carnosus muscle and approaching the collecting LVs was observed (Fig 5Li to 5Liv).

## Discussion

Three main results were obtained in the present study. First, the deep medial system, the superficial medial system, a connection between the superficial and deep medial lymphatic systems, and the superficial lateral system were identified (Fig 3A and 3B). These lymphatic systems were similar to those identified in previous studies. A connection between the superficial and deep medial lymphatic systems has been discovered in mice (route3 in Fig 3A) [20], and it was also observed in the rats in the present study. Second, three types of detours were identified after lymphatic flow blockage (Fig 4A). Lastly, detours were located in the fatty layer above the panniculus carnosus muscle, and their lumens were wide. There were LVs with narrow lumen above the panniculus carnosus muscle in normal tissue.

A connection between the superficial and deep medial systems has already been discovered in mice by Nakajima *et al*. [20], and we found this route in rats. The route is believed to transport the lymph from the inguinal region to the deep lymphatic system. Similarly, in rats, a connection between the superficial and deep lymphatic systems was considered to act as a hindlimb lymphatic drainage route. In rats, other routes found in the present study, i.e., the deep medial system (route1 in Fig 3A), superficial medial system (route2 in Fig 3A), and superficial lateral system (route5 in Fig 3B), have been found in previous studies [16, 17].

In the present study, the right hindlimb was more swollen than the left one at 3 days after lymphatic flow blockage. Nonetheless, there was no difference in the size of the right and left hindlimbs after 30 days. In other words, the lymphedema disappeared. It was considered that detours, the deep medial system, and a connection between the superficial and deep medial lymphatic systems could be responsible for the edema reduction. Several previous studies have also described edema resolution over time. Kwon et al. [21], who resected popliteal LN in mice reported that the swelling of the hindlimb on the surgical side reached a maximum on postoperative day 6 and then resolved. Similarly, Frueh et al. [22] collected tissue from the hindlimb of the mouse 3 and 10 days after LN dissection and reported that the dilated subcutaneous lymph vessels normalized, and the swelling decreased.

The detour to the LV near the midline of the abdomen (detour b in Fig 4A) and the detour to the contralateral inguinal LN (detour c in Fig 4A) were involved in the hindlimb lymphatic transport. The detour to the contralateral inguinal LN has been investigated in a previous study [16]. Detours to the LV near the midline have been observed in rats, but not mentioned explicitly.

Histological assessment of the detours showed that they were formed by the pre-collecting LVs, as observed in the study by Asano et al. [15]. In the histology of the detour to the left inguinal LN, the LV was seen above the panniculus carnosus muscle in the image farthest from the left inguinal LN. In the image closer to the left inguinal LN, the LV was seen to penetrate the panniculus carnosus muscle, and then the LV was seen below the panniculus carnosus muscle. Furthermore, the LV approached the collecting LV below the panniculus carnosus muscle. In addition, detours were not accompanied by blood vessels like collecting LVs. The LVs with these characteristics were found in the same location as that of the pre-collecting LVs with narrow lumina in normal rats. Additionally, there were two possible reasons for the appearance of the detours. The first is due to the spread of the lymphatic network formed by capillary LVs in the dermis, pre-collecting LVs in the subcutaneous tissues connecting with the capillary LVs, and collecting LVs in the subcutaneous tissues on the epimysium or along the deep blood vessels. The lymphatic system comprises a vast network that extends throughout the dermis and subcutaneous tissues [10]. Therefore, when some routes are blocked, it is likely that the lymph is carried through other network paths to deeper LVs. The second is due to changes in the fluid pressure gradients in the LVs. The direction of the lymphatic flow is determined by fluid pressure gradients [23]. When lymphatic flow is blocked, high outflow pressure is applied to the surrounding LVs. This pressure is supposed to trigger an influx of lymph into the LVs that are not normally involved in the transport of hindlimb lymph. Regarding changes in the lymphatic routes after lymphatic flow blockage, Yamaji *et al*. observed lymphatic flow changes when they resected the popliteal LNs of rats [24]. Similarly, Asano *et al*. reported that after ligation of the LVs, a detour appeared to straddle the original pathway at the ligation site [15]. In addition, Kwon *et al*. stated that the detour that developed immediately after LN dissection disappeared when other detours appeared [21]. For these reasons, changes in the lymphatic network and fluid pressure within the LVs due to the blockage of lymphatic flow may influence subsequent lymphatic route changes.

In clinical implementation, identifying detours may help in the development of a new method of SLD. SLD is time-consuming because it involves whole body massage the whole body to promote lymphatic drainage. In this study, we found that the pathway that occurs after lymph flow blockage is different from the normal one. Therefore, we considered the possibility of incorporating lymphatic fluid induction into the massage method to promote lymphatic uptake into the pre-collecting LVs. This is thought to be able to shorten the care time and contribute to reducing the patient’s self-care burden.

This study had some limitations. First, it was cross-sectional and did not follow subsequent changes in the lymphatic pathways. In addition, LV wall and luminal diameter measurements, as well as the ability to excrete ICG, were not investigated; therefore, their details were not clarified. Furthermore, the results were for Slc: Wistar rats and may not be directly applicable to other animals.

## Conclusions

The rats in the present study had four hindlimb lymphatic systems: the deep medial system, the superficial medial system, a connection between the superficial and deep medial lymphatic systems, and the superficial lateral system. Three types of detours, namely the detour of the lateral abdomen, the detour to the LV near the midline of the abdomen, and the detour to the contralateral inguinal LN, were identified after lymphatic flow blockage. Detours were located in the fatty layer above the panniculus carnosus muscle and their lumina were wide. The histology suggested that the detour was a dilated pre-collecting LV. These findings may contribute to the development of a new method of SLD.

## Supporting information

S1 Fig Detours confirmed in each of the eight rats. ICG images show the detours observed in each rat. The top of the figure is the cephalic side, the bottom is the caudal side, and the fluorescence of the ICG is observed from the body’s surface. The detours are tortuous and avoid the LN dissection area. The light green circles show the site of inguinal LN dissection. The white arrow heads indicate detours.
(TIF)

S1 Table Circumference and volume of the hindlimbs before iliac LN dissection and at 3 and 30 days inguinal and popliteal LN dissection in each rat. (PDF)
