# Supplementary figures and images for: Comparison of normal hindlimb lymphatic systems in rats with detours present after lymphatic flow blockage
Source: PLoS One. 2021 Dec 13;16(12):e0260404. doi: 10.1371/journal.pone.0260404 (PMC8668128; doi:10.1371/journal.pone.0260404)

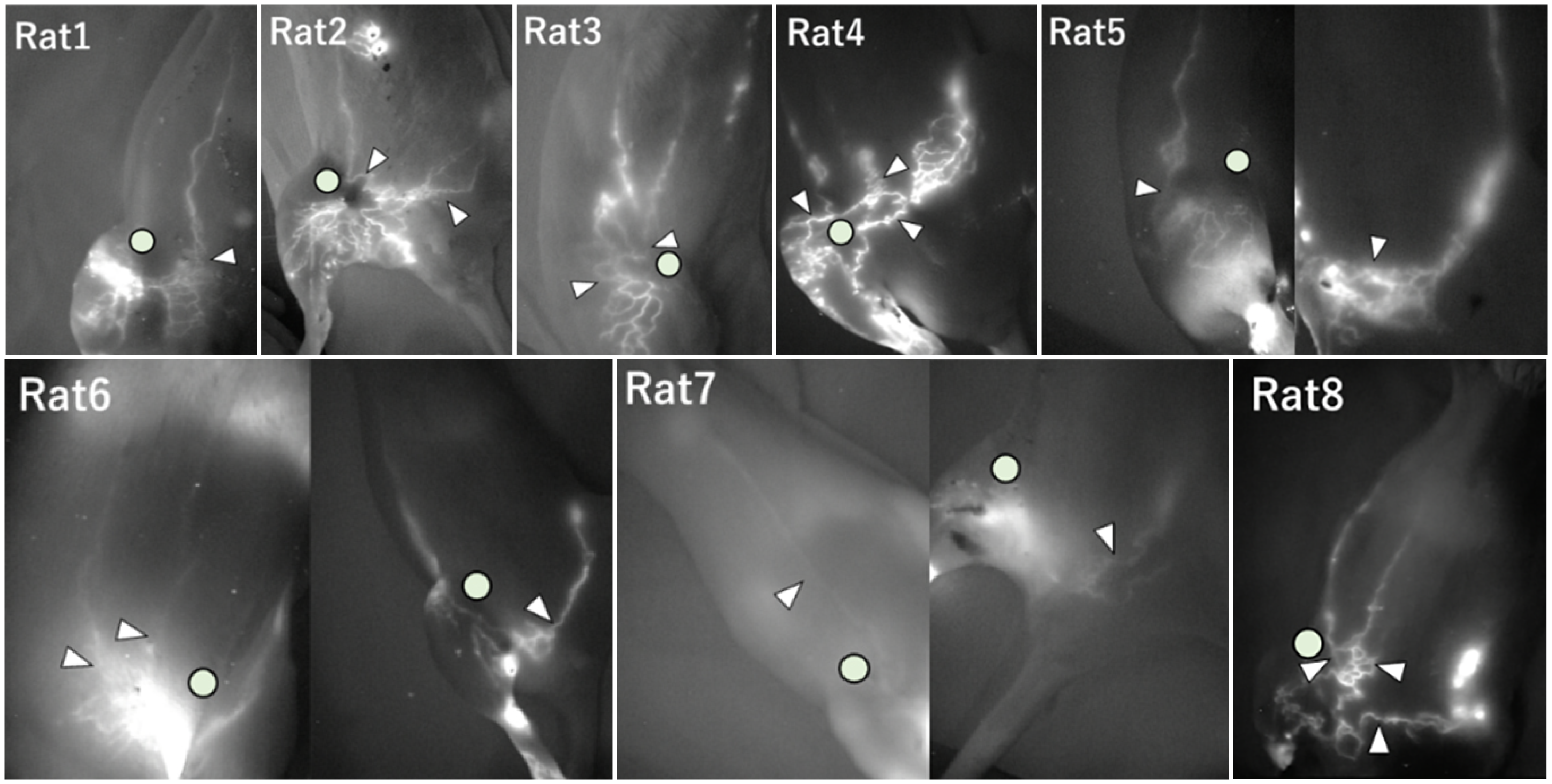

Supplement: S1 Fig — ICG images show the detours observed in each rat. The top of the figure is the cephalic side, the bottom is the caudal side, and the fluorescence of the ICG is observed from the body’s surface. The detours are tortuous and avoid the LN dissection area. The light green circles show the site of inguinal LN dissection. The white arrow heads indicate detours. (TIF) [file pone.0260404.s001.tif]
