# Supplementary material for: Comparison of normal hindlimb lymphatic systems in rats with detours present after lymphatic flow blockage
Source: PLoS One. 2021 Dec 13;16(12):e0260404. doi: 10.1371/journal.pone.0260404 (PMC8668128; doi:10.1371/journal.pone.0260404)
Supplement: S1 Table — (PDF) [file pone.0260404.s002.pdf]

Circumference (㎝)

| | | | Rat1 | Rat2 | Rat3 | Rat4 | Rat5 | Rat6 | Rat7 | Rat8 |
|---|---|---|---|---|---|---|---|---|---|---|
| Before | R | 1 | 7.7 | 7.5 | 8.4 | 8.2 | 6.9 | 7.1 | 6.9 | 7.8 |
| | | 2 | 8.2 | 7.3 | 8.2 | 8.5 | 6.9 | 7.4 | 7.0 | 7.6 |
| | | 3 | 7.9 | 7.3 | 8 | 8.4 | 6.8 | 7.6 | 7.3 | 7.7 |
| | | Average | 7.9 | 7.4 | 8.3 | 8.4 | 6.9 | 7.4 | 7.1 | 7.5 |
| | L | 1 | 7.5 | 7.0 | 8.4 | 8.1 | 6.7 | 7.5 | 7.1 | 8.1 |
| | | 2 | 7.5 | 7.4 | 8.4 | 8.4 | 6.9 | 7.3 | 7.2 | 7.7 |
| | | 3 | 7.7 | 7.3 | 8.3 | 8.2 | 6.8 | 7.6 | 7.1 | 8.0 |
| | | Average | 7.6 | 7.2 | 8.4 | 8.3 | 6.8 | 7.5 | 7.1 | 7.9 |
| 3 days | R | 1 | 8.7 | 8 | 8.2 | 10.2 | 7.6 | 7.5 | 7.2 | 8.6 |
| | | 2 | 9 | 8.1 | 8.6 | 9.8 | 7.6 | 7.6 | 7.3 | 8.7 |
| | | 3 | 8.5 | 7.6 | 8.6 | 9.8 | 7.6 | 8.4 | 7.3 | 8.5 |
| | | Average | 8.7 | 7.9 | 8.5 | 10 | 7.6 | 8.1 | 7.3 | 8.6 |
| | L | 1 | 7.9 | 7.6 | 8.7 | 8.6 | 7.9 | 7.9 | 6.8 | 7.7 |
| | | 2 | 7.9 | 7.7 | 9.4 | 8.6 | 6.4 | 8.3 | 6.9 | 7.5 |
| | | 3 | 8.0 | 7.0 | 8.7 | 8.8 | 7.1 | 8.4 | 6.9 | 7.7 |
| | | Average | 7.9 | 7.4 | 8.9 | 8.7 | 7.1 | 8.2 | 6.9 | 7.6 |
| 30 days | R | 1 | 7.1 | 7.4 | 8.2 | 9.0 | 7.3 | 7.5 | 7.2 | 7.5 |
| | | 2 | 7 | 8.2 | 8.9 | 9.2 | 7.2 | 7.6 | 7.2 | 7.5 |
| | | 3 | 7 | 8.6 | 8.6 | 8.7 | 7.3 | 8.4 | 7.1 | 7.5 |
| | | Average | 7.0 | 8.1 | 8.6 | 9.0 | 7.3 | 7.8 | 7.2 | 7.5 |
| | L | 1 | 7.5 | 8 | 8.2 | 8.5 | 6.4 | 9 | 7.4 | 9 |
| | | 2 | 7.6 | 8.7 | 8 | 9.1 | 6.8 | 7.7 | 7.2 | 8.5 |
| | | 3 | 7.7 | 7.4 | 8.2 | 8.8 | 7.5 | 7.6 | 7.4 | 9.5 |
| | | Average | 7.6 | 8.0 | 8.1 | 8.8 | 6.9 | 8.1 | 7.3 | 9.0 |

Volume (ml)

| | | | Rat1 | Rat2 | Rat3 | Rat4 | Rat5 | Rat6 | Rat7 | Rat8 |
|---|---|---|---|---|---|---|---|---|---|---|
| Before | R | 1 | 10.1 | 6.2 | 6.1 | 9.3 | 4.4 | 5.4 | 1.7 | 3.8 |
| | | 2 | 8.2 | 6.6 | 6.1 | 8.6 | 4.1 | 4.5 | 2.5 | 3.8 |
| | | 3 | 9.5 | 8.1 | 5.5 | 8.5 | 4.6 | 5.3 | 3.0 | 4.9 |
| | | Average | 9.2 | 7.0 | 5.9 | 8.8 | 4.4 | 5.1 | 2.4 | 4.2 |
| | L | 1 | 8.3 | 7.4 | 5.3 | 7.7 | 4.6 | 6.1 | 1.4 | 3.8 |
| | | 2 | 9.1 | 7.2 | 6.8 | 7.3 | 3.5 | 4.2 | 2.3 | 4.6 |
| | | 3 | 11.0 | 6.9 | 6.7 | 8.0 | 3.7 | 3.9 | 2.6 | 4.2 |
| | | Average | 9.5 | 7.2 | 6.3 | 7.6 | 3.9 | 4.7 | 2.1 | 4.2 |
| 3 days | R | 1 | 11.3 | 7.9 | 5.8 | 13.4 | 4.4 | 5.4 | 1.6 | 4.3 |
| | | 2 | 11.1 | 7.4 | 6.6 | 13.4 | 4.7 | 4.5 | 3.2 | 4.7 |
| | | 3 | 9.6 | 9.3 | 8.1 | 13.4 | 4.5 | 5.7 | 2.6 | 5.6 |
| | | Average | 10.7 | 8.2 | 6.8 | 13.4 | 4.5 | 5.2 | 2.4 | 4.8 |
| | L | 1 | 9.4 | 7.4 | 7.3 | 12.7 | 3.7 | 3.6 | 1.4 | 3.8 |
| | | 2 | 7.8 | 7.6 | 5.5 | 11.9 | 4.1 | 4.0 | 2.0 | 4.3 |
| | | 3 | 11.3 | 7.5 | 7.2 | 12.3 | 4.0 | 4.1 | 3.0 | 4.6 |
| | | Average | 9.5 | 7.5 | 6.7 | 12.3 | 4.0 | 3.9 | 2.1 | 4.3 |
| 30 days | R | 1 | 10.8 | 8.1 | 6.5 | 8.4 | 4.9 | 3.8 | 3.9 | 3.7 |
| | | 2 | 9.3 | 9.0 | 6.1 | 7.4 | 4.8 | 4.7 | 3.5 | 4.0 |
| | | 3 | 9.5 | 8.5 | 6.7 | 10.6 | 3.8 | 5.1 | 2.8 | 5.0 |
| | | Average | 9.9 | 8.5 | 6.5 | 8.8 | 4.5 | 4.6 | 3.4 | 4.2 |
| | L | 1 | 11.2 | 8.6 | 7.2 | 6.9 | 4.2 | 2.9 | 3.2 | 4.0 |
| | | 2 | 9.0 | 9.6 | 6.8 | 7.4 | 4.1 | 3.6 | 3.1 | 4.8 |
| | | 3 | 10.1 | 7.8 | 6.0 | 7.4 | 4.0 | 4.4 | 4.3 | 4.1 |
| | | Average | 10.1 | 8.7 | 6.7 | 7.3 | 4.1 | 3.6 | 3.5 | 4.3 |
